# Di-μ-aqua-bis­{triaqua­[5-(1-oxopyridin-4-yl)tetra­zol-1-ido]sodium}

**DOI:** 10.1107/S1600536810052566

**Published:** 2010-12-24

**Authors:** Jing Dai, Xin-Yuan Chen

**Affiliations:** a Ordered Matter Science Research Center, College of Chemistry and Chemical Engineering, Southeast University, Nanjing 210096, People’s Republic of China

## Abstract

In the title compound, [Na_2_(C_6_H_4_N_5_O)_2_(H_2_O)_8_], the Na^I^ atom is in a distorted octahedral environment defined by six O atoms, one from the 5-(1-oxopyridin-4-yl)tetra­zolide anion and five from water mol­ecules. Two water mol­ecules act as bridging ligands, resulting in the formation of dimeric units organized around inversion centers. In the organic anion, the pyridine and tetra­zole rings are nearly coplanar, forming a dihedral angle of 4.62 (1)°. The dimeric units and organic anions are connected by O—H⋯O and O—H⋯N hydrogen bonds, leading to the formation of a three-dimensional network.

## Related literature

For tetra­zole derivatives, see: Zhao *et al.* (2008 ▶); Fu *et al.* (2008 ▶, 2009 ▶). For the structures and properties of related compounds, see: Fu *et al.* (2007 ▶, 2009 ▶); Fu & Xiong (2008 ▶).
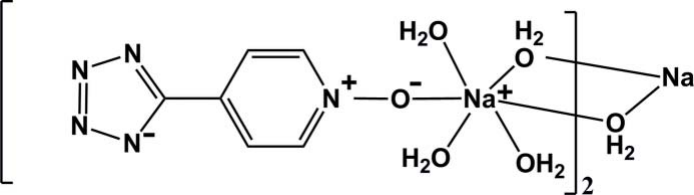


## Experimental

### 

#### Crystal data

[Na_2_(C_6_H_4_N_5_O)_2_(H_2_O)_8_]
*M* *_r_* = 514.39
Triclinic, 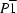

*a* = 6.887 (2) Å
*b* = 7.5200 (15) Å
*c* = 12.258 (5) Å
α = 78.16 (4)°
β = 83.42 (4)°
γ = 66.68 (3)°
*V* = 570.2 (3) Å^3^
*Z* = 1
Mo *K*α radiation
μ = 0.16 mm^−1^
*T* = 298 K
0.25 × 0.15 × 0.10 mm

#### Data collection

Rigaku Mercury2 diffractometer
Absorption correction: multi-scan (*CrystalClear*; Rigaku, 2005 ▶) *T*_min_ = 0.913, *T*_max_ = 1.000
5833 measured reflections
2594 independent reflections
1933 reflections with *I* > 2σ(*I*)
*R*_int_ = 0.028

#### Refinement

*R*[*F* ^2^ > 2σ(*F* ^2^)] = 0.041
*wR*(*F* ^2^) = 0.108
*S* = 1.05
2594 reflections
154 parameters
H-atom parameters constrained
Δρ_max_ = 0.23 e Å^−3^
Δρ_min_ = −0.25 e Å^−3^

### 

Data collection: *CrystalClear* (Rigaku, 2005 ▶); cell refinement: *CrystalClear*; data reduction: *CrystalClear*; program(s) used to solve structure: *SHELXS97* (Sheldrick, 2008 ▶); program(s) used to refine structure: *SHELXL97* (Sheldrick, 2008 ▶); molecular graphics: *ORTEPIII* (Burnett & Johnson, 1996 ▶), *ORTEP-3 for Windows* (Farrugia, 1997 ▶) and *PLATON* (Spek, 2009 ▶); software used to prepare material for publication: *SHELXTL* (Sheldrick, 2008 ▶).

## Supplementary Material

Crystal structure: contains datablocks I, global. DOI: 10.1107/S1600536810052566/dn2636sup1.cif

Structure factors: contains datablocks I. DOI: 10.1107/S1600536810052566/dn2636Isup2.hkl

Additional supplementary materials:  crystallographic information; 3D view; checkCIF report

## Figures and Tables

**Table 1:** Hydrogen-bond geometry (Å, °)

*D*—H⋯*A*	*D*—H	H⋯*A*	*D*⋯*A*	*D*—H⋯*A*
O1*W*—H1*WA*⋯N2^i^	0.85	1.98	2.832 (2)	178
O1*W*—H1*WB*⋯N4^ii^	0.86	1.97	2.817 (2)	167
O2*W*—H2*WA*⋯O3*W*^iii^	0.85	2.02	2.857 (2)	169
O2*W*—H2*WB*⋯N3^ii^	0.87	2.02	2.878 (2)	169
O3*W*—H3*WA*⋯O4*W*^iv^	0.85	1.93	2.754 (2)	163
O3*W*—H3*WB*⋯O1^iv^	0.85	2.07	2.836 (2)	150
O4*W*—H4*WA*⋯O1*W*^v^	0.86	1.96	2.812 (2)	172
O4*W*—H4*WB*⋯O1^iv^	0.85	1.95	2.7233 (19)	151

Symmetry codes: (i) 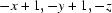; (ii) 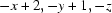; (iii) 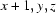; (iv) 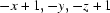; (v) 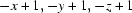.

**Fig. 1.**
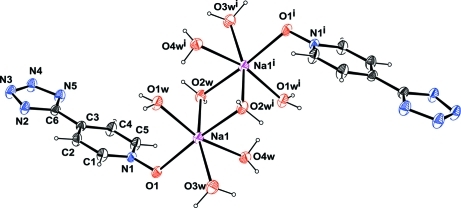
A view of the title compound with the atomic numbering scheme. Displacement ellipsoids were drawn at the 30% probability level. [Symmetry code: (i) -x+1, -y+1, -z+1]

**Fig. 2.**
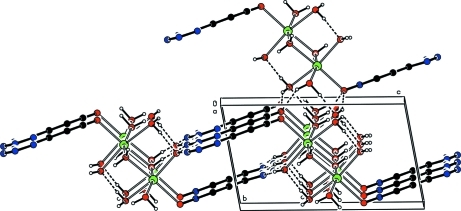
The crystal packing of the title compound, showing the three-dimensional hydrogen-bonded chain. H atoms not involved in hydrogen bonding (dashed line) have been omitted for clarity.

## supplementary crystallographic information

### Comment

Tetrazole compounds attracted more attention as phase transition dielectric materials for its application in micro-electronics, memory storage. With the purpose of obtaining phase transition crystals of tetrazol-pyridine compounds, its interaction with various metal ions has been studied and a series of new materials have been elaborated with this organic molecule (Zhao *et al.*, 2008; Fu *et al.*, 2008; Fu *et al.*, 2007; Fu & Xiong 2008). In this paper, we describe the crystal structure of the title compound, tetraaquabis[5-(1-oxopyridin-4-yl)tetrazol-1-ide]sodium(I).

In the title compound, the asymmetric unit is composed of one organic anion, four H_2_O molecules and one Na^+^ cation. The Na^I^ center, with slightly distorted octahedral geometry, is surrounded by six oxygen atoms. Two water molecules act as abridging ligand, resulting in the formation of dimeric unit (Fig. 1) organized around inversion center. In the organic anion, the tetrazole N atoms are deprotonated. The pyridine and tetrazole rings are nearly coplanar and only twisted from each other by a dihedral angle of 4.62 (1)°. The geometric parameters of the tetrazole rings are comparable to those in related molecules (Zhao *et al.*, 2008; Fu *et al.*, 2009).

In crystal structure, the intermolecular hydrogen bonds are formed by all H atoms of the water molecules with tetrazole N atoms or with the O atoms. The complex dinuclear cation units, [Na_2_(H_2_O)_8_]^2+^, are linked in the crystal through O–H···O H-bonds into broad infinite cation-cation sheet parallel to the (0 0 1) plane. The two-dimensional sheets are linked by organic anions through O—H···N and O—H···O H-bonds into a three-dimensional framework (Table 1 and Fig.2).

### Experimental

A mixture of 4-(1*H*-tetrazol-5-yl)pyridine 1-oxide (0.4 mmol) and NaOH (0.4 mmol), ethanol (1 ml) and a few drops of water sealed in a glass tube was maintained at 373 K. Colorless needle crystals suitable for X-ray analysis were obtained after 3 days.

While the permittivity measurement shows that there is no phase transition within the temperature range (from 100 K to 400 K), and the permittivity is 8.4 at 1 MHz at room temperature.

### Refinement

All H atoms attached to C atoms were fixed geometrically and treated as riding with C–H = 0.93 Å (aromatic) with *U*_iso_(H) = 1.2*U*_eq_(C). H atoms of water molecule were located in difference Fourier maps and included in the subsequent refinement using restraints (O-H= 0.85 (1)Å and H···H= 1.40 (2)Å) with U_iso_(H) = 1.5U_eq_(O). In the last cycles of refinement, they were treated as iding on their parent O atoms.

### Crystal data

**Table:**

[Na_2_(C_6_H_4_N_5_O)_2_(H_2_O)_8_]	*Z* = 1
*M**_r_* = 514.39	*F*(000) = 268
Triclinic, *P*1	*D*_x_ = 1.498 Mg m^−^^3^
Hall symbol: -P 1	Mo *K*α radiation, λ = 0.71073 Å
*a* = 6.887 (2) Å	Cell parameters from 2594 reflections
*b* = 7.5200 (15) Å	θ = 3.0–27.5°
*c* = 12.258 (5) Å	µ = 0.16 mm^−^^1^
α = 78.16 (4)°	*T* = 298 K
β = 83.42 (4)°	Needle, colourless
γ = 66.68 (3)°	0.25 × 0.15 × 0.10 mm
*V* = 570.2 (3) Å^3^	

### Data collection

**Table:**

Rigaku Mercury2 diffractometer	2594 independent reflections
Radiation source: fine-focus sealed tube	1933 reflections with *I* > 2σ(*I*)
graphite	*R*_int_ = 0.028
Detector resolution: 13.6612 pixels mm^-1^	θ_max_ = 27.5°, θ_min_ = 3.0°
ω scans	*h* = −8→8
Absorption correction: multi-scan (*CrystalClear*; Rigaku, 2005)	*k* = −9→9
*T*_min_ = 0.913, *T*_max_ = 1.000	*l* = −15→15
5833 measured reflections	

### Refinement

**Table:**

Refinement on *F*^2^	Primary atom site location: structure-invariant direct methods
Least-squares matrix: full	Secondary atom site location: difference Fourier map
*R*[*F*^2^ > 2σ(*F*^2^)] = 0.041	Hydrogen site location: inferred from neighbouring sites
*wR*(*F*^2^) = 0.108	H-atom parameters constrained
*S* = 1.05	*w* = 1/[σ^2^(*F*_o_^2^) + (0.047*P*)^2^ + 0.0948*P*] where *P* = (*F*_o_^2^ + 2*F*_c_^2^)/3
2594 reflections	(Δ/σ)_max_ < 0.001
154 parameters	Δρ_max_ = 0.23 e Å^−^^3^
0 restraints	Δρ_min_ = −0.25 e Å^−^^3^

### Special details

**Table:**

Geometry. All esds (except the esd in the dihedral angle between two l.s. planes) are estimated using the full covariance matrix. The cell esds are taken into account individually in the estimation of esds in distances, angles and torsion angles; correlations between esds in cell parameters are only used when they are defined by crystal symmetry. An approximate (isotropic) treatment of cell esds is used for estimating esds involving l.s. planes.
Refinement. Refinement of *F*^2^ against ALL reflections. The weighted *R*-factor *wR* and goodness of fit *S* are based on *F*^2^, conventional *R*-factors *R* are based on *F*, with *F* set to zero for negative *F*^2^. The threshold expression of *F*^2^ > σ(*F*^2^) is used only for calculating *R*-factors(gt) *etc*. and is not relevant to the choice of reflections for refinement. *R*-factors based on *F*^2^ are statistically about twice as large as those based on *F*, and *R*- factors based on ALL data will be even larger.

### Fractional atomic coordinates and isotropic or equivalent isotropic displacement parameters (Å^2^)

**Table:**

	*x*	*y*	*z*	*U*_iso_*/*U*_eq_	
Na1	0.44822 (10)	0.33639 (10)	0.43816 (5)	0.0346 (2)	
O1	0.61821 (19)	0.08957 (19)	0.31881 (9)	0.0378 (3)	
O1W	0.33072 (19)	0.59396 (19)	0.28360 (10)	0.0414 (3)	
H1WA	0.2225	0.6181	0.2472	0.062*	
H1WB	0.4285	0.6085	0.2368	0.062*	
O2W	0.74752 (18)	0.43381 (18)	0.43714 (9)	0.0370 (3)	
H2WA	0.8677	0.3598	0.4637	0.056*	
H2WB	0.7715	0.5053	0.3761	0.056*	
O3W	0.1735 (2)	0.1911 (2)	0.49613 (11)	0.0454 (3)	
H3WA	0.2095	0.0977	0.4599	0.068*	
H3WB	0.2038	0.1404	0.5635	0.068*	
O4W	0.6251 (2)	0.12583 (19)	0.61122 (10)	0.0413 (3)	
H4WA	0.6486	0.2025	0.6463	0.062*	
H4WB	0.5445	0.0774	0.6532	0.062*	
N1	0.7291 (2)	0.1343 (2)	0.22842 (11)	0.0308 (3)	
N2	1.0244 (2)	0.3306 (2)	−0.15939 (12)	0.0392 (4)	
N3	1.1872 (2)	0.3602 (3)	−0.21981 (12)	0.0429 (4)	
N4	1.3389 (2)	0.3266 (3)	−0.15339 (12)	0.0443 (4)	
N5	1.2806 (2)	0.2732 (2)	−0.04794 (12)	0.0419 (4)	
C1	0.6427 (3)	0.1983 (3)	0.12848 (14)	0.0390 (4)	
H1	0.5044	0.2109	0.1220	0.047*	
C2	0.7555 (3)	0.2455 (3)	0.03546 (14)	0.0383 (4)	
H2	0.6926	0.2900	−0.0334	0.046*	
C3	0.9619 (3)	0.2278 (2)	0.04267 (13)	0.0303 (4)	
C4	1.0458 (3)	0.1597 (3)	0.14777 (15)	0.0433 (5)	
H4	1.1842	0.1445	0.1566	0.052*	
C5	0.9284 (3)	0.1147 (3)	0.23857 (14)	0.0423 (5)	
H5	0.9875	0.0698	0.3084	0.051*	
C6	1.0873 (3)	0.2775 (2)	−0.05454 (13)	0.0307 (4)	

### Atomic displacement parameters (Å^2^)

**Table:**

	*U*^11^	*U*^22^	*U*^33^	*U*^12^	*U*^13^	*U*^23^
Na1	0.0354 (4)	0.0374 (4)	0.0307 (4)	−0.0152 (3)	0.0012 (3)	−0.0040 (3)
O1	0.0434 (7)	0.0472 (8)	0.0282 (6)	−0.0272 (6)	0.0108 (5)	−0.0046 (5)
O1W	0.0358 (7)	0.0603 (9)	0.0308 (6)	−0.0255 (6)	−0.0033 (5)	0.0017 (6)
O2W	0.0314 (6)	0.0441 (7)	0.0317 (6)	−0.0143 (5)	0.0023 (5)	−0.0005 (5)
O3W	0.0418 (7)	0.0474 (8)	0.0421 (7)	−0.0136 (6)	−0.0051 (6)	−0.0024 (6)
O4W	0.0440 (7)	0.0448 (8)	0.0398 (7)	−0.0241 (6)	0.0021 (6)	−0.0053 (6)
N1	0.0339 (8)	0.0364 (8)	0.0252 (7)	−0.0189 (6)	0.0044 (6)	−0.0039 (6)
N2	0.0349 (8)	0.0588 (10)	0.0273 (7)	−0.0248 (7)	0.0011 (6)	−0.0015 (7)
N3	0.0389 (9)	0.0609 (11)	0.0308 (8)	−0.0263 (8)	0.0051 (6)	−0.0009 (7)
N4	0.0383 (9)	0.0659 (11)	0.0330 (8)	−0.0292 (8)	0.0043 (7)	−0.0021 (7)
N5	0.0346 (8)	0.0631 (11)	0.0327 (8)	−0.0274 (8)	0.0014 (6)	−0.0019 (7)
C1	0.0291 (9)	0.0599 (12)	0.0326 (9)	−0.0242 (8)	−0.0018 (7)	−0.0032 (8)
C2	0.0343 (9)	0.0577 (12)	0.0264 (9)	−0.0235 (8)	−0.0037 (7)	−0.0012 (8)
C3	0.0301 (8)	0.0354 (9)	0.0279 (8)	−0.0169 (7)	0.0012 (6)	−0.0032 (7)
C4	0.0327 (9)	0.0675 (14)	0.0332 (10)	−0.0278 (9)	−0.0039 (7)	0.0029 (9)
C5	0.0359 (10)	0.0660 (13)	0.0273 (9)	−0.0266 (9)	−0.0052 (7)	0.0037 (8)
C6	0.0293 (8)	0.0360 (9)	0.0278 (8)	−0.0157 (7)	0.0010 (6)	−0.0025 (7)

### Geometric parameters (Å, °)

**Table:**

Na1—O1W	2.3665 (19)	N1—C1	1.336 (2)
Na1—O2W^i^	2.4305 (17)	N1—C5	1.339 (2)
Na1—O1	2.4408 (18)	N2—C6	1.334 (2)
Na1—O2W	2.4437 (15)	N2—N3	1.340 (2)
Na1—O4W	2.486 (2)	N3—N4	1.311 (2)
Na1—O3W	2.5080 (17)	N4—N5	1.337 (2)
Na1—Na1^i^	3.4684 (16)	N5—C6	1.330 (2)
O1—N1	1.3344 (17)	C1—C2	1.371 (2)
O1W—H1WA	0.8508	C1—H1	0.9300
O1W—H1WB	0.8595	C2—C3	1.386 (2)
O2W—Na1^i^	2.4305 (18)	C2—H2	0.9300
O2W—H2WA	0.8506	C3—C4	1.386 (2)
O2W—H2WB	0.8674	C3—C6	1.464 (2)
O3W—H3WA	0.8460	C4—C5	1.365 (2)
O3W—H3WB	0.8469	C4—H4	0.9300
O4W—H4WA	0.8571	C5—H5	0.9300
O4W—H4WB	0.8501		
			
O1W—Na1—O2W^i^	89.59 (6)	Na1—O3W—H3WA	104.0
O1W—Na1—O1	92.55 (6)	Na1—O3W—H3WB	100.9
O2W^i^—Na1—O1	174.05 (5)	H3WA—O3W—H3WB	107.3
O1W—Na1—O2W	86.19 (6)	Na1—O4W—H4WA	105.9
O2W^i^—Na1—O2W	89.27 (5)	Na1—O4W—H4WB	111.5
O1—Na1—O2W	96.41 (5)	H4WA—O4W—H4WB	107.4
O1W—Na1—O4W	163.27 (5)	O1—N1—C1	120.51 (14)
O2W^i^—Na1—O4W	83.50 (6)	O1—N1—C5	119.42 (14)
O1—Na1—O4W	95.86 (6)	C1—N1—C5	120.07 (15)
O2W—Na1—O4W	78.53 (6)	C6—N2—N3	104.47 (14)
O1W—Na1—O3W	109.49 (6)	N4—N3—N2	109.39 (14)
O2W^i^—Na1—O3W	85.24 (5)	N3—N4—N5	109.73 (14)
O1—Na1—O3W	88.81 (5)	C6—N5—N4	104.48 (14)
O2W—Na1—O3W	163.30 (5)	N1—C1—C2	120.75 (16)
O4W—Na1—O3W	85.17 (6)	N1—C1—H1	119.6
O1W—Na1—Na1^i^	87.03 (5)	C2—C1—H1	119.6
O2W^i^—Na1—Na1^i^	44.79 (4)	C1—C2—C3	120.88 (16)
O1—Na1—Na1^i^	140.86 (5)	C1—C2—H2	119.6
O2W—Na1—Na1^i^	44.48 (4)	C3—C2—H2	119.6
O4W—Na1—Na1^i^	77.33 (5)	C4—C3—C2	116.46 (16)
O3W—Na1—Na1^i^	128.08 (5)	C4—C3—C6	120.99 (15)
N1—O1—Na1	118.00 (10)	C2—C3—C6	122.55 (15)
Na1—O1W—H1WA	124.0	C5—C4—C3	120.99 (17)
Na1—O1W—H1WB	115.4	C5—C4—H4	119.5
H1WA—O1W—H1WB	108.1	C3—C4—H4	119.5
Na1^i^—O2W—Na1	90.73 (5)	N1—C5—C4	120.86 (16)
Na1^i^—O2W—H2WA	109.3	N1—C5—H5	119.6
Na1—O2W—H2WA	125.5	C4—C5—H5	119.6
Na1^i^—O2W—H2WB	105.5	N5—C6—N2	111.93 (15)
Na1—O2W—H2WB	116.3	N5—C6—C3	123.24 (15)
H2WA—O2W—H2WB	106.5	N2—C6—C3	124.82 (15)

Symmetry codes: (i) −*x*+1, −*y*+1, −*z*+1.

### Hydrogen-bond geometry (Å, °)

**Table:**

*D*—H···*A*	*D*—H	H···*A*	*D*···*A*	*D*—H···*A*
O1W—H1WA···N2^ii^	0.85	1.98	2.832 (2)	178.
O1W—H1WB···N4^iii^	0.86	1.97	2.817 (2)	167.
O2W—H2WA···O3W^iv^	0.85	2.02	2.857 (2)	169.
O2W—H2WB···N3^iii^	0.87	2.02	2.878 (2)	169.
O3W—H3WA···O4W^v^	0.85	1.93	2.754 (2)	163.
O3W—H3WB···O1^v^	0.85	2.07	2.836 (2)	150.
O4W—H4WA···O1W^i^	0.86	1.96	2.812 (2)	172.
O4W—H4WB···O1^v^	0.85	1.95	2.7233 (19)	151.

Symmetry codes: (ii) −*x*+1, −*y*+1, −*z*; (iii) −*x*+2, −*y*+1, −*z*; (iv) *x*+1, *y*, *z*; (v) −*x*+1, −*y*, −*z*+1; (i) −*x*+1, −*y*+1, −*z*+1.
